# Auricular Acupuncture with Laser

**DOI:** 10.1155/2013/984763

**Published:** 2013-06-26

**Authors:** Regina Round, Gerhard Litscher, Frank Bahr

**Affiliations:** ^1^Frank Bahr Research Group “Auriculomedicine and Pharmacopuncture”, Stronach Research Unit for Complementary and Integrative Laser Medicine, Research Unit of Biomedical Engineering, Anesthesia and Intensive Care Medicine, and the TCM Research Center Graz, Medical University of Graz, Auenbruggerplatz 29, 8036 Graz, Austria; ^2^European Academy for TCM, 81245 Munich, Germany

## Abstract

Auricular acupuncture is a method which has been successfully used in various fields of medicine especially in the treatment of pain relief. The introduction of lasers especially low-level lasers into medicine brought besides the already existing stimulation with needles and electricity an additional technique to auricular acupuncture. This literature research looks at the historical background, the development and the anatomical and neurological aspects of auricular acupuncture in general and auricular laser acupuncture in detail. Preliminary scientific findings on auricular acupuncture with laser have been described in detail and discussed critically in this review article. The results of the studies have shown evidence of the effect of auricular laser acupuncture. However, a comparison of these studies was impossible due to their different study designs. The most important technical as well as study parameters were described in detail in order to give more sufficient evidence and to improve the quality of future studies.

## 1. Introduction

Today, there are many publications on acupuncture but only a few on auricular acupuncture. When you search for the term “acupuncture” in the scientific database PubMed (http://www.pubmed.gov/), more than 18,300 publications are found, while only 836 publications show up when you search for the term “auricular acupuncture.”

The reason for this can be found in history. Acupuncture, being a part of Traditional Chinese Medicine (TCM), has been practiced over thousands of years. From the beginning, Chinese Medicine was passed on from one generation to the next. Therefore, many books have been written—some of them more than 2,100 years ago. 

Auricular acupuncture has also been practiced for about 2,500 years, but it was not passed on the same way as TCM was. Only about 60 years ago auricular acupuncture experienced its revival through the French physician named Paul Nogier. 

Due to his ongoing research and successful treatments—especially in the field of pain management—auricular acupuncture developed into a distinct treatment system of its own. Currently, auricular acupuncture is used for acute as well as chronic pain associated with sciatica, osteoarthritis, headache, knee arthroscopy, hip fracture, hip arthroplasty, and even cancer [1]. 

As a result of the technological progress not only the range of treatment has expanded but also have the methods used for stimulating the acupuncture points developed from metal needles over electroacupuncture to laser acupuncture [2]. 

Research shows that laser acupuncture is still in its early stage of development and up to now only a small amount of different studies have been published. 

The results of these studies, however, show that there is high potential in the field of laser acupuncture. Among others there are two main reasons why laser acupuncture should be considered as an alternative treatment to needle acupuncture: first it offers a noninvasive treatment for children or patients who are afraid of needles and second the laser treatment requires less time.

## 2. Classical Acupuncture

Classical acupuncture has its origin in TCM and it is believed to have been practiced for about 2,500 years. It is described as a medical treatment by inserting needles into the skin at specific points on the body. 

Acupuncture is based on the principle of Qi. Qi can be described as a vital life force. According to TCM, you inherit Qi when you are born and you obtain Qi by breathing and eating during your lifetime. Qi flows in energetic pathways or channels in the human body—the so-called meridians. They form a meridian system which partially relates to the internal organs and their physiological and pathological conditions. Along these meridians, energy comes to the surface through a series of acupuncture points. They serve as tunnels or access routes to the deeper meridian system. A dysfunction of the Qi can be restored by stimulating these acupuncture points [3–5]. 

It should not remain unmentioned that in 1991 a mummy named Iceman or Ötzi was found in the Ötztaler Alps in South Tyrol. Scientists believed that the Iceman was about 5,200 years old. His body showed tattoos on the locations of classical acupuncture points. Due to the strong evidence that was found Dorfer et al. [6] hypothesized that a medical system similar to Chinese acupuncture already existed in Central Europe over 5,200 years ago. This leads to the conclusion that acupuncture originated in the Eurasian continent and is nearly 2,000 years older than it was believed to be. 

## 3. Auricular Acupuncture

For a better understanding of auricular acupuncture, we will look at its history and development from two different perspectives:

- *ancient auricular acupuncture* referring to the time before the 20th century and up to the 1950s,
- *modern auricular acupuncture* referring to the time when Paul Nogier rediscovered auricular acupuncture.

Again this term can be divided into

- the *Chinese system* of auricular acupuncture and
- the *Western or European system* of auricular acupuncture, which further developed into auriculotherapy and auriculomedicine [7].

### 3.1. Ancient Auricular Acupuncture

#### 3.1.1. Definition

Ancient auricular acupuncture is a treatment based on the stimulation of acupuncture points on the auricle. These points were believed to be connected to the meridians and used empirically for certain treatments mainly for pain relief. They, however, were not part of an acupuncture system limited to the ear but part of a system covering the whole body [8, 9]. 

#### 3.1.2. History

There is only little evidence from the past to prove which culture ear acupuncture originates from. According to Oleson [7] and Chen [8] a relationship between the ear, the meridians, and the viscera has already been mentioned in the most famous and over 2,100-year old book *Huang Di Nei Jing*—the *Yellow Emperor's Classic of Internal Medicine*. Some of the auricular points which are referred to in this and other books are still used today. They, however, have no connection to the meridians and no logical order. 

There has also been historical reference to the use of auricular treatments by Hippocrates, the “father of the western medicine” and paragon of ancient physicians, who lived around 460 BC. During his studies in Egypt, Hippocrates learned about the treatment of impotence and how to facilitate ejaculation by practising phlebotomy on the veins of the posterior surface of the ear. He kept on practising and teaching this method at his medical school on the island of Kos. Today, scientists assume that the therapeutic effect was not necessarily caused by phlebotomy itself but simply by stimulating the ear. 

Another example is Mediterranean sailors who wore their golden earrings on the central lobule of the ear not only for decoration but also for improving their eyesight [7–11].

In 1636 Lusitanus Zactaus, a Portuguese doctor, described the treatment of low back pain and sciatica by cauterizing the earlap. About sixty years later, in 1717, the Italian doctor Antonio Maria Valsalva found new areas on the ear to relieve dental pain by cauterization. He described this in his famous book *De Aure Humana Tractatus*. In 1810 Professor Ignazio Colla of Parma reported a patient who claimed that after being stung by a bee in the antihelix, all the pain in his legs was gone immediately. From around 1850 onwards, there was a big hype about ear cauterization. It was mainly used as relief from dental pain or during dental extraction entirely. Sometimes it was even used to prevent dental extraction. Dysfunctions of the facial nerve were also treated this way. Due to scientific inexplicability, however, the methods began to be forgotten [9, 10]. 

### 3.2. Modern Auricular Acupuncture 

#### 3.2.1. Definition

According to Gori and Firenzuoli [9], modern auricular acupuncture is defined as “a diagnostic and treatment system based on normalizing the body's dysfunction by stimulating acupuncture points on the external ear.” It is not based on TCM, but on the presumption of Paul Nogier that a somatotopic organization of the body is represented on the human auricle. 

#### 3.2.2. History

It was not until 1950 when the French physician and later founder of auriculotherapy Dr. Paul Nogier discovered peculiar scars on the antihelix of his patient, who had been successfully treated for sciatic pain syndrome. These scars were caused by cauterization. Presuming it was something new, he started research and discovered that the pain ceased within a couple of hours or sometimes even minutes after being cauterized. 

**Figure 1:**
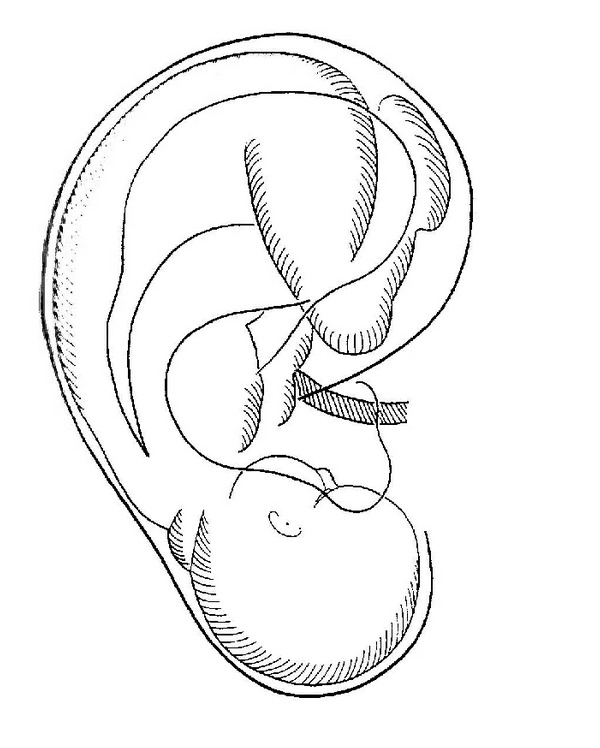
Inverted fetus. Modified from [10].

Following a series of his clinical studies, Paul Nogier replaced cauterizing with needling the acupuncture points. Despite the lack of scientific validation, Paul Nogier kept on investigating this phenomenon. In his research he theorized that a somatotopy, as already known of the sensory and motor cortices of the brain, could also be presented on the ear. This resulted in his discovery of the body's anatomy displaying itself on the ear as an inverted fetus (see Figure 1).

In 1956 Dr. Niboyet, France's most famous acupuncturist at the time, took notice of Paul Nogier's work and invited him to speak at the Congress of the Société Mediterranéenne in Marseille. The presentation was later published in the German acupuncture journal “Deutsche Zeitschrift für Akupunkteure.” Eventually, Paul Nogier's discovery found its way to China where a group of Chinese acupuncturists conducted a study on over 2000 patients which allowed them to verify his findings. In 1959 an article about French auricular acupuncture was published in a Chinese journal called “Popular Medicine” in which Paul Nogier was credited for his discovery. At the same year the Chinese acknowledged Paul Nogier as “the Father of Auricular Acupuncture” [7, 10, 12].

### 3.3. The Chinese and the Western System of Auricular Acupuncture

Like his Chinese colleagues, Paul Nogier carried on investigating. While Paul Nogier continued his work by looking at the auricular points from an anatomical point of view, Chinese scientists developed the Chinese system by looking at the functional connection between the auricular acupuncture points and their effects on the body. 

In 1966, Paul Nogier discovered that the radial artery pulse showed a reaction to the stimulation of the auricle. Assuming that this response involved the ear and the heart, he called this response “Reflexe Auriculocardiaque.” Later when he realized that this response was caused by the autonomic nervous system, he changed its name to vascular autonomic signal (VAS). This reflex or signal can be explained as a vascular reaction to the stimulation of the skin. With this unique vascular reflex auricular therapy advanced into auricular medicine, a system for advanced diagnosis and treatment.

In the late 1950s Paul Nogier introduced a small number of 42 auricular acupuncture points to Chinese scientists. Only 20 years later, in the late 1970s, Chinese acupuncturists had already increased the number to over 1,000 points most of which were empirically found. 

In 1982, the World Health Organization (WHO) launched international working groups with the aim to facilitate teaching, research, and clinical practice of auricular acupuncture all over the world. In 1990, a standardization of auricular acupuncture was established by introducing 3 criteria. An auricular point had to have an international and a common name, its therapeutic value had to be proven, and its location on the auricle had to be generally accepted. Thirty nine auricular points fulfilled all 3 criteria; 36 points failed one or more of the criteria. Finding an agreement on these issues was a very important step in the development of auricular acupuncture. Due to different approaches of French and Chinese auricular acupuncturists, there is still a lot of discrepancy on certain auricular points and their mappings [8, 10, 13, 14]. 

## 4. Paul Nogier 

Paul Nogier was born in Lyon in 1908. He was the son of a tenured professor at the Medical University of Lyon. After 3 years of studying physics, Paul Nogier went on to study medicine.

In 1938, he established his general medicine practice in Lyon where he also practiced homeopathy, manipulative medicine, and body acupuncture. To pursue these methods, Paul Nogier started a monthly study session with his students in 1942, which he kept going until he died in 1996. In 1969, he published his first book called *Traité d'Auriculothérapie*. 

Furthermore, he received some of France's most honorable awards, and in 1990 the WHO honored him for his great contribution in medicine and recognized him as the founder of auricular therapy and auricular medicine. Only a few months before his death in 1996 the *Ècole Internationale Paul Nogier *was founded [12, 15]. 

Raphael Nogier, Paul's son, pursued his father's work and described him as “a man of innovative thoughts and productive action who listened to his patients, respected what they had to say and thoroughly investigated their maladies. He tirelessly examined his patients from Monday morning to Saturday evening” [7]. 

## 5. The Auricle 

### 5.1. Microsystem of the Ear

A microsystem is the projection of the whole body in its function and structure on certain parts of the body. In addition to the auricle microsystems can also be found on the scalp, the feet, the hands, and the iris. 

Thanks to Paul Nogier's rediscovery, auricular acupuncture has developed into one of the most commonly used and explored microsystems in the last 60 years [15, 16]. 

### 5.2. Anatomy of the Auricle

The ear is divided into 3 anatomic parts: the external ear (lat.: auris externa), the middle ear (lat.: auris media), and the internal ear (lat.: auris interna). 

The external ear consists of the pinna or auricle (lat.: auricular), the external auditory canal (lat.: meatus acusticus externus), and the tympanic membrane (lat.: membrane tympanica).

In auricular acupuncture the most important anatomic structure is the auricle. The ear cartilage (lat.: cartilage auricularis) provides a supporting framework for the auricle and gives the auricle its funnel shape, while the ear lobe (lat.: lobulus auricularis) has no elastic cartilaginous part at all. 

The outer part of the ear cartilage is called the helix. The helix crus divides the concha, the deepest point of the auricle, into the cymbia conchae or hemiconcha superior and the cavity of conchae or hemiconcha inferior. 

At the opposite of the helix, one finds the antihelix. In between lies the triangular fossa. The sulcus between the antihelix and helix, is called the scapha or scaphoid fossa. Together with the helix it disembogues in the ear lobe. 

**Figure 2:**
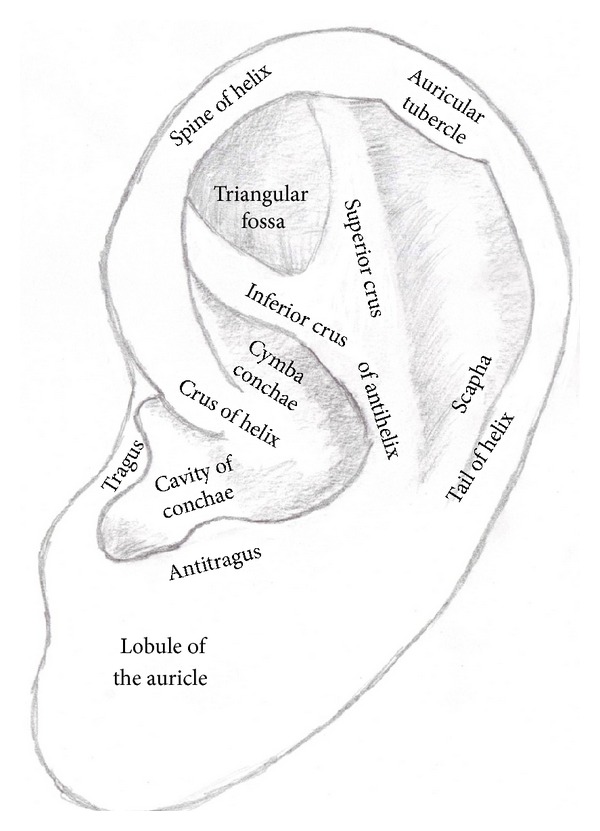
Anatomy of the auricle.

The cartilaginous bead that lies in front of the external auditorial canal is called tragus. Opposite of it is the antitragus. Between the tragus and the antitragus lies the incisura intertragica (see Figure 2) [10, 17, 18]. 

The posterior auricle can be divided into 5 main parts of the back of the ear: the posterior groove behind the antihelix, the posterior lobe, the posterior concha, the posterior triangle behind the triangular fossa, and the posterior periphery behind the scapha and the helix [19]. 

### 5.3. Innervation of the Auricle (See Figure 3)

The auricle is innervated by spinal and cranial nerves (CN). The facial nerve is responsible for the motoric innervation of the outer ear muscle. 

Responsible for the sensitive innervation are the greater auricle nerve (GAN), the lesser occipital nerve, the auriculotemporal nerve—the third division and mandibular branch of the trigeminal nerve—and the auricular branches of the vagus nerve (CN X), the glossopharyngeal nerve (CN IX), and the facial nerve (CN VII) [18]. 

The studies of He et al., Ueno et al. [20, 21], and Peuker and Filler [17] do not coincide on the areas of innervation. 

According to He et al. and Ueno et al. [20, 21] the greater auricle nerve (GAN) innervates both surfaces of the lower part of the auricle whereas Peuker and Filler [17] find additional innervation of the GAN in the tail of the helix and the scapha.

He et al. and Ueno et al. [20, 21] suggest that the lesser occipital nerve supplies the skin of the upper and back parts of the auricle with fibers. Peuker and Filler [17] do not mention this nerve in their studies. 

According to He et al. and Ueno et al. [20, 21] the auriculotemporal nerve innervates the anterosuperior and the anteromedial areas of the auricle (see also Table 1). This involves the crus and the upper part of helix body, the antihelix, the triangular fossa, and the tragus. Peuker and Filler [17] found the auriculotemporal nerve solely in the crus and the upper part of the helix.

The auricle branch of the vagus nerve (ABVN) supplies the concha, mainly the cavity of the conchae, and most of the region around the external auditorial canal according to He et al. and Ueno et al. [20, 21]. Peuker and Filler [17] found the ABVN also in the concha but mainly in the cymbia of the conchae and also in the antihelix.

## 6. Modern Auricular Acupuncture

### 6.1. Somatotopy

**Figure 4:**
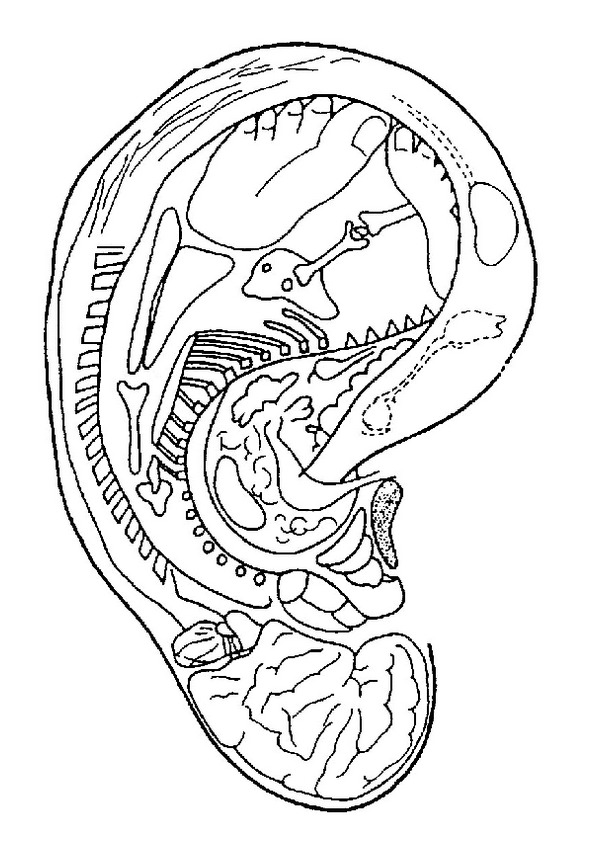
Somatotopy. Modified from [10].

Paul Nogier's discovery of the inverted fetus being projected on the auricle is the basis of modern auricular acupuncture. As it is shown in Figure 4 the head with the brain structures are represented on the ear lobe; the spine runs along the antihelix. The inner organs are represented in the concha. The legs and the arms are represented towards the upper rim of the auricle. This somatotopic representation is often seen as a homunculus like it is known of the sensory cortex in the brain [16, 22]. 

### 6.2. Embryological Aspects

**Figure 5:**
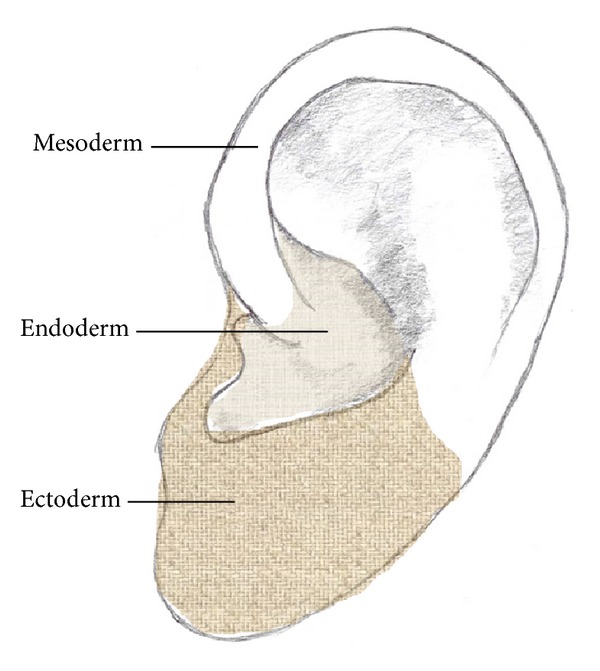
Auricular embryology.

The somatotopic mapping also reveals an embryological pattern of the organs. As you can see in Figure 5, organs originating from the ectoderm are presented on the ear lobe and the tragus, organs originating from the endoderm on the concha, and mesodermal organs on the remaining part of the auricle [16]. 

### 6.3. Neurological Aspects

The mechanism of auricular acupuncture is believed to work through the autonomic nervous system. By stimulating the auricle, the information travels through sympathetic and parasympathetic nerve fibers from the ear to the brain and from the brain through the spinal cord to specific areas in the body [16, 19]. 

Heine [23] stated in his survey that auricular acupuncture points are dot-like structures with a diameter of one-tenth millimetres at the most. These structures consist of a combination of collagen and elastic fibers which are pervaded by nerve endings, arterioles, venules, and capillaries. 

According to Soliman and Frank [16], the vagal nerve carries the parasympathetic fibers and the branch of the trigeminal nerve carries the sympathetic fibers to the reticular formation where the information gets distributed to the corresponding brain structures. 

So far there is no evidence in the literature that auricular acupuncture has a direct influence on the sympathetic system. However, numerous studies have shown that auricular acupuncture influences the parasympathetic activity, thus, also the autonomic nervous system which can have an impact on the cardiovascular system, the endocrine system, the respiratory and gastrointestinal system, the urinary system, and in the treatment of epilepsy and depression or even an anti-inflammatory effect [19, 20, 24–31]. 

With a 75% share of all parasympathetic fibers, the vagus nerve has the highest influence on the parasympathetic system [32]. 

The afferent fibers of the vagus nerve synapse in structures of the medulla in particular in the nucleus tractus solitarius (NTS), the nucleus of the spinal tract of the trigeminal nerve, medial reticular formation of the medulla, the area postrema, the dorsal motor nucleus of the vagus, and the nucleus ambiguus. The majority of the vagal fibers lead into the NTS. Most of the information that is received by the NTS is spread to various areas of the brain such as the hypothalamus, the central nucleus of the amygdala, and other nuclei in the brainstem. 

The efferent fibers of the vagus nerve lead to the heart, lungs, stomach, intestines, liver, pancreas, and kidneys [20, 33]. 

Despite the high amount of parasympathetic fibers of the vagus nerve, the auricle branch is the only peripheral branch of the vagus nerve. Thus, He et al. [20] theorized that auricular vagal stimulation can alter the autonomic as well as the central nervous system via the concha to the NTS and further on to the corresponding structures of the brain. They suggested the existence of an “auriculovagal afferent pathway.”

## 7. Detection of the Auricular Acupuncture Points 

French and Chinese physicians suggested already in the 1970s that the auricle can not only be used for therapy but also for the diagnosis of dysfunctions in the body [22, 34–36]. 

The survey of Oleson et al. [22] investigated the theory by examining patients with musculoskeletal pain. In 75.2% of the cases there was an accordance between the conventional medical diagnosis and the auricular diagnosis. It is believed that the reaction of the ear is caused again through neurological pathways. In normal conditions, the ear is “electrically silent,” but under pathological conditions the ear turns “electrically active” [16]. 

The inspection and palpation of the auricle are primary steps for the detection of “reactive points.” Abnormalities like swelling, blushing, desquamation, or tenderness can be signs of pathological conditions in the body. 

**Figure 6:**
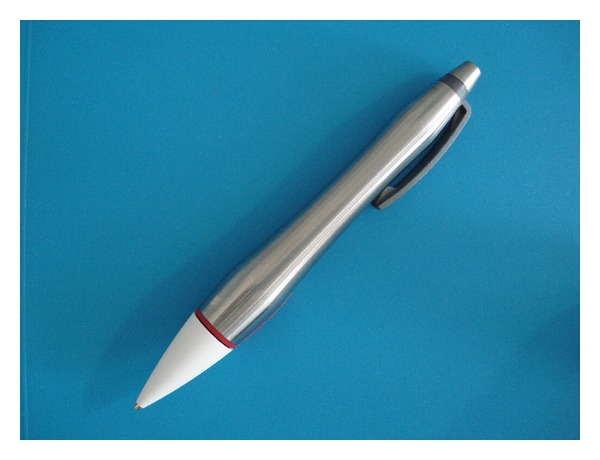
Point finder [10].

Mechanical and electrical point finders (Figure 6) are also used for a more precise detection of acupuncture points. Mechanical point finders depend very much on the sensitivity of the patients which can often lead to distorted results, whereas electrical point finders supply more objective results by measuring the electrical skin resistance. Recently, point finders with laser have been applied for the identification of “reactive acupuncture points” as well [15, 22]. 

Besides inspection, palpation, and point detectors, the vascular autonomic signal (VAS) is also used for the finding of auricular points. 

## 8. Vascular Autonomic Signal (VAS) 

Paul Nogier discovered this signal in 1966 assuming that there was a connection between the ear and the heart. Later, he realized that it was a response of the generalized autonomic nervous system. 

**Figure 7:**
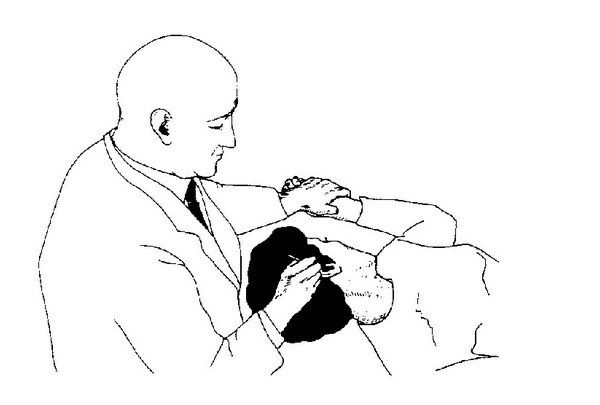
Detection of VAS. Modified from [10].

The VAS is a vasculocutaneous reflex that can be felt like a stationary wave of the pulse on the arterial wall (compare Figure 7). This pulse is the result of cardiac output and its rebound as the blood piles up against the arterioles and capillaries. It is a response and adjustment of the nervous system. Any stimulation of the skin by light, laser, heat, or touch can provoke the VAS. 

In Europe, the VAS became another method used for finding “reactive” auricular points. Hereby, practitioners use the radial artery pulse for its detection. VAS also allows the identification of treatable points which are not painful but still show a dysfunction of the body. 

While in the past practitioners used their thumb to palpate the VAS, nowadays it can also be detected with bidirectional Doppler ultrasonography [11]. 

## 9. Methods of Stimulating the Ear Points 

### 9.1. Acupressure

Auricular acupressure is a noninvasive method that stimulates the acupuncture points by applying pressure using fingers, knuckles, or dull objects like magnet beads or vaccaria seeds.

### 9.2. Needle Acupuncture

There are different methods of stimulating the ear points. The most common method is acupuncture. Hereby needles are used for the stimulation of the auricle. In former days people used fish bones, sharp stones, or bamboo clips as acupuncture material. Later, these materials were replaced with gold or silver. Today, the needles are made of high tensile stainless steel and coated in gold or silver. Gold needles are used for reducing (supplying) and silver needles are used for reinforcing (drainage). On the back of the ear they are applied the other way round. The needles stay in place for about 20–45 minutes.

Sometimes after an acupuncture treatment semipermanent needles are attached to certain acupuncture points to enhance the effect of the treatment. These needles are solely made of high tensile stainless steel and stay in the ear for an average time of one week [10, 37]. 

### 9.3. Electro Acupuncture

Electroacupuncture is a new modern technique of controlled electrical stimulation on the ear. Hereby, small electrical currents are applied to needles which have been inserted at specific acupuncture points. 

The stimulation can either be on low frequency (2 Hz) or high frequency (100 Hz) depending on the pathological issue. 

A new auricular electroacupuncture device called P-Stim was invented in the early 1990s by the Austrian physician Dr. Szeles and has been subject of many studies mainly on the treatment of chronic and acute pain since then [38, 39]. 

### 9.4. Laser Acupuncture

Laser acupuncture is a noninvasive method that stimulates auricular acupuncture points by applying laser light. It will be further discussed in the following chapters. 

## 10. Adverse Reactions of Auricular Acupuncture

Adverse reactions happen very rarely in auricular acupuncture. So far, there have been reports neither on life-threatening reactions nor on irreversible or serious adverse events after acupuncture. Minor events can be local pain, local bleeding, or infections after needle acupuncture. There have been nine documented cases of perichondritis and one case of chondritis since 1980. In the worst case these diseases can lead to chondronecrosis or even deformation of the auricle. Out of these ten cases, eight patients received permanent needle acupuncture. Proper disinfection and regular inspection of permanent needles can help to avoid such side effects [40]. 

It has been suggested that patients with diabetes mellitus, immunosuppressed patients, or weakened patients suffering from chronic diseases should not receive permanent needle treatment [41]. 

## 11. Important Points in Auricular Acupuncture 

As mentioned earlier, there is a huge amount of auricular points used in the Chinese as well as in the Western system. In the following, the most commonly used and researched points in auricular acupuncture will be introduced.

## 12. Shenmen

**Figure 8:**
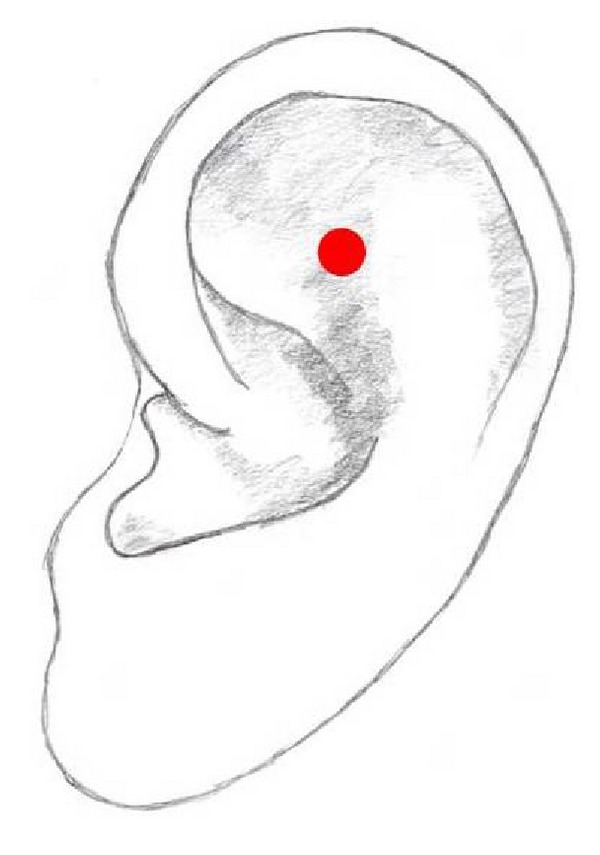
Shenmen [7, 63].

Shenmen, meaning “spirit gate,” is located at the apex of the triangular fossa (Figure 8). This point is derived from the Chinese system but is also used in the Western system. Generally, it is used in combination with other auricular points. It is also the most commonly used point in auricular acupuncture mainly for the treatment of pain, stress, anxiety, and depression as well as alcohol or drug abuse, cessation of smoking, and weight loss [1, 19, 20, 25, 30, 42–62]. 

## 13. Heart Point

**Figure 9:**
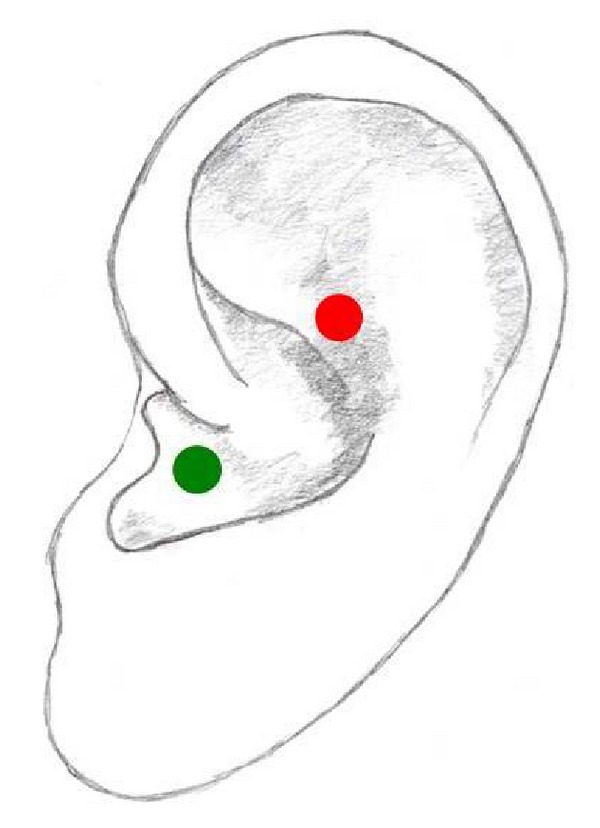
Heart point of the Western system (red), Heart point of the Chinese system (green) [7, 63].

There are two different locations of the Heart point in auricular acupuncture. According to the Western system, the Heart point is located in the antihelix whereas in the Chinese system it is situated in the hemiconcha inferior (Figure 9). 

In most of the studies, the Chinese Heart point and its considered effect on the cardiovascular system and vagal activity have been investigated [25–27, 31, 64–66]. 

In their survey on advanced auricular acupuncture, Frank and Soliman [67] explained the shifting of the location of the Heart point and the Chinese Shenmen according to the three phases which depend on the stage of illness. 

Phase 1, representing the original map of the inverted fetus, stands for normal physiology or acute pathology. In phase 2, the inverted fetus is represented in an upright position on the map and stands for degenerative conditions. Phase 3 shows the fetus in a transverse position and stands for subacute and chronic conditions. 

With this advanced approach and the Western point of view of the representation of the anatomy and the organs on the auricle, Frank and Soliman [67] showed that Shenmen correlates with the Western auricular points of the spleen in phase 1, the thalamus in phase 2, and the liver in phase 3. They also showed that through the shift the location of the Chinese and the Western Heart point coincide in phase 2. 

## 14. Thalamus Point

**Figure 10:**
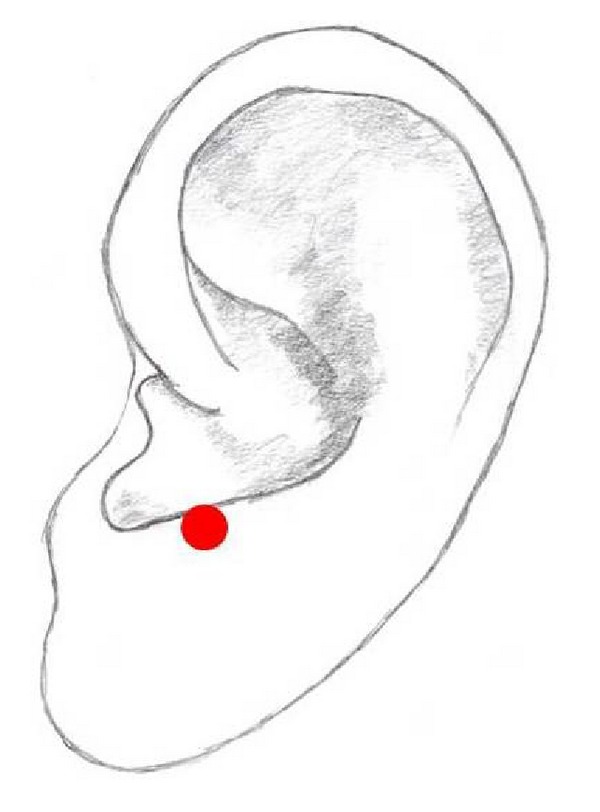
Thalamus point [7, 63].

The Thalamus point is situated on the lower edge of the antitragus (Figure 10). This point is mainly used in combination with Shenmen and the Lung point in pain therapy [1, 53]. 

## 15. Point Zero 

**Figure 11:**
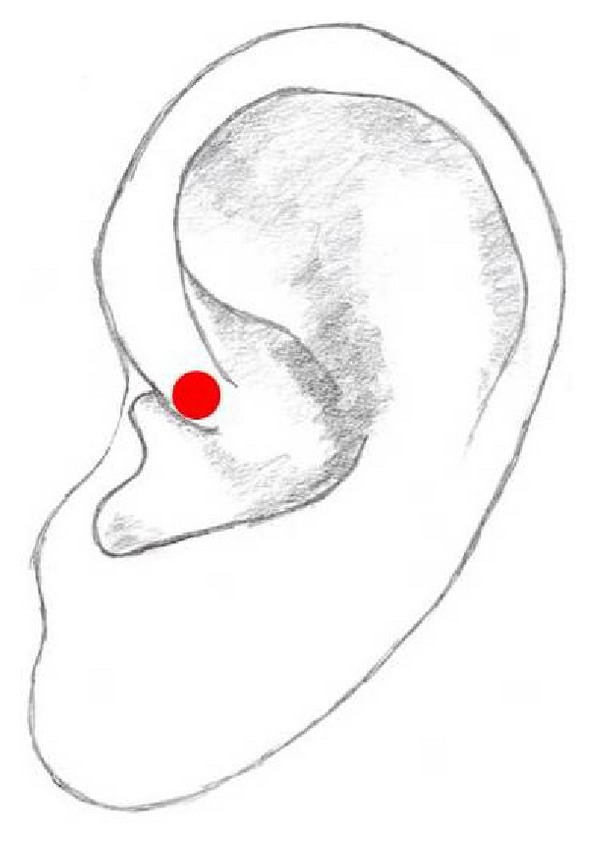
Point Zero [7, 63].

Point Zero is located on a notch in the crus of the helix (Figure 11). It is one of the most important points of the auricle and was first described by Paul Nogier. He named this point “Point Zero” to express its function as a balancing point responsible for the homeostatic in the body in analogy to the “center of the body” according to the tenets of TCM [10, 19]. 

This point is used in the treatment of pain relief in combination with other auricular acupuncture points including Shenmen [53]. 

## 16. Lung Points 

**Figure 12:**
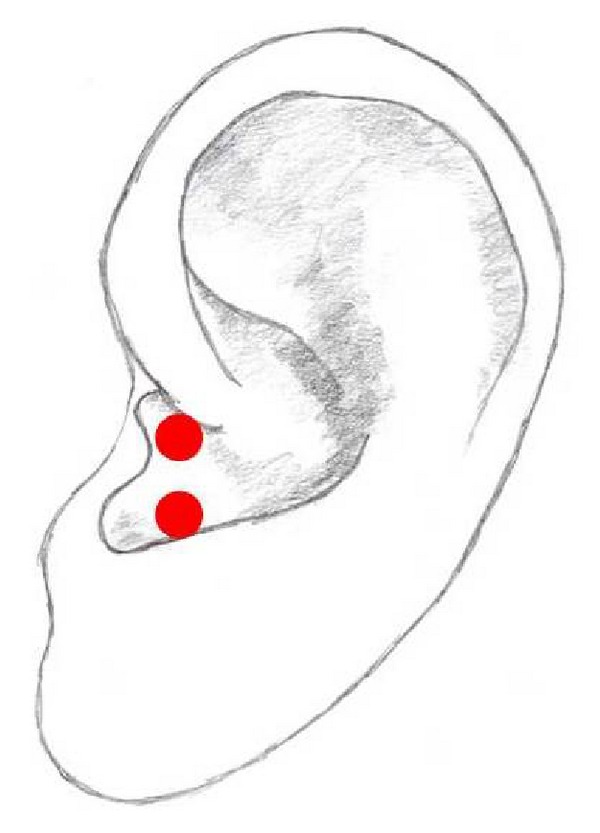
Lung points [7, 63].

There are 2 Lung points (Figure 12). Both are situated in the hemiconcha inferior, representing the right or the left side of the lung. They are also two of the most commonly used auricular acupuncture points. In most studies only one point is stimulated. The Lung points are mainly used for the treatment of alcohol or drug dependency and pain relief especially after surgery. Studies show that auricular acupuncture might have an impact on the autonomic nervous system [1, 25, 28, 31, 43–45, 48, 51, 55–57]. 

## 17. More Details on the Auricular Acupuncture Points

Studies which investigated the effects of auricular acupuncture on pathological conditions often used a combination of auricular acupuncture points and other auricular or even body acupuncture points. 

Studies on pain therapy after surgeries, for example, used Shenmen and one of the Lung points as basic points in combination with the auricular acupuncture point corresponding to the treated area, for example, the point for the knee joint after a knee surgery [55, 57]. 

Studies on drug and alcohol dependencies used a combination of auricular points suggested by the National Acupuncture Detoxification Association (NADA) including Shenmen, Sympathetic point, Kidney point, Liver point, and Lung point [43, 44, 48]. 

Studies investigating the effects of auricular acupuncture on the autonomic nervous system tested either the Heart point or the Lung point individually or in comparison to body acupuncture points. Both points have their locations in the concha, the area which is innervated mainly by the vagus nerve [25–28, 64, 65, 68]. There are also studies investigating the effect of Shenmen on anxiety compared to other auricular acupuncture points or body acupuncture points [58, 61]. 

Many studies provide poor information about the names or the exact locations of the treated auricular acupuncture points; mostly they only refer to the anatomical areas that had been treated. This lack of information is one of the problems in comparing the studies and makes it difficult to prove the effectiveness of auricular acupuncture [24, 47, 49, 69–73]. 

## 18. Recent Studies on Auricular Acupuncture

This section covers recent studies of auricular acupuncture which were conducted either by acupressure, needling, or electro acupuncture. Studies on auricular laser acupuncture will be treated later on separately.

In former days, auricular acupuncture was mainly used for the treatment of sciatica and low back pain. After Paul Nogier's discovery of the microsystem on the ear, numerous clinical and experimental studies on the effects of auricular acupuncture have been carried out mainly in Europe, Asia, and America. Studies show that besides the already known success in treating low back pain many other diseases can be cured by using auricular acupuncture.

A review of Chen [46] showed that auricular acupuncture offers a vast field of applications. Fifty six kinds of illnesses including diseases of the respiratory tract, the circulatory system, the digestive system, the urinary system and the nervous system, diseases in the fields of otolaryngology, ophthalmology, dermatology, paediatrics, and gynaecology as well as acute abdomen, obesity, drinking, and smoking cessation were discussed in these studies. This review included retrospective as well as randomized controlled studies. Auricular acupuncture was effective in most cases especially in the treatment of hypertension, acute pain of the digestive system, dermatological diseases—like psoriasis, urticarial, or acne—and obesity. Due to the small amounts of treated people and randomized controlled (RCT) studies, the quality of this review is poor but still it shows that auricular acupuncture has the potential of being effective in many fields of medicine.

### 18.1. Pain

There are many studies on pain management with auricular acupuncture. It has shown its effectiveness on acute as well as chronic pain. Low back pain, headache, and pain caused by osteoarthritis were treated with auricular acupuncture. It was even used for postoperative pain control [1, 39]. 

Studies have shown that auricular acupuncture can be effective especially in the treatment of postoperative pain which would further result in a decrease of analgesic requirement. More studies should be conducted for more reliable and conclusive scientific evidence [55–57, 72, 74]. 

### 18.2. Obesity

There is evidence that auricular acupuncture can be used to achieve weight loss. Hsu et al. [49] ran a randomized controlled study with 60 obese women. These women were treated with auricular acupuncture over a period of 3 months. Although there was no change in body weight, body mass index, and waist circumference, there was a significant difference in the levels of obesity-related hormones. Particularly the hormone ghrelin showed an increase and the hormone leptin showed a decrease after 3 months of treatment. Previous studies have shown that obese people have a lower level of ghrelin than people with normal weight and that an increase of this level can be associated with weight loss. 

### 18.3. Anxiety

Studies on anxious patients before operations or dental treatments and on older patients with postoperative anxiety have given evidence of the effectiveness of auricular acupuncture in these fields. The mechanism is still inconclusive, but scientists believe that auricular acupuncture might have an impact on the release of neurotransmitters such as serotonin which helps in the regulation of anxiety [42, 58, 61, 75, 76]. 

However, there is still no evidence on a successful treatment of patients with generalized anxiety disorder or anxiety neurosis [76]. 

### 18.4. Cardiovascular System

Recent studies on rats showed that auricular acupuncture regulates the cardiovascular function by activating baroreceptor sensitive neurons in the nucleus tractus solitarius (NTS). The arterial pressure and the heart rate were significantly reduced by auricular acupuncture [26, 77]. 

Another Chinese publication reported a decrease in systolic as well as diastolic pressure in patients with hypertension after electric pulse stimulation. The total effective rate had been 90.7% [68]. 

### 18.5. Insomnia

There is evidence that auricular acupuncture can also be effective in cases of insomnia. A combination of auricular acupressure with routine care was more efficient than solely auricular acupressure. However, due to methodological limitations and inconclusive results further evidence and research have yet to be considered [47, 52, 78]. 

### 18.6. Dermatological Conditions

Only a few studies have been implemented on psoriasis vulgaris. So far, there is one survey that shows a therapeutic effect of auricular acupuncture in combination with a decoction of TCM [79]. 

Auricular acupuncture has also been used for the treatment of verruca plana or flat warts. This randomized work showed a statistically better outcome in the treatment group than in the control group [80]. 

However, in both cases further investigations need to be considered to have more scientific evidence on the treatment of psoriasis vulgaris as well as verruca plana. 

### 18.7. Opiate Addiction and Alcohol Abuse

The euphoric feeling of cocaine is assumed to be triggered by blocking the reuptake of the neurotransmitters noradrenalin, serotonin, and mainly dopamine. Hereby cocaine acts as an indirect dopamine agonist [48]. 

Auricular acupuncture is believed to have its effect by stimulating the vagus nerve at one of the Lung points on the auricle. This leads to a stimulation of the hypothalamus, which leads to the release of serotonin and further to an activation of methionine encephalin which inhibits the release of *γ*-aminobutyric acid (GABA). The inhibition of GABA causes an increase in dopamine. Hypothetically this mechanism reduces cravings and hereby supports the patient on his path to self-recovery [48]. 

Besides a Lung point the NADA determined further auricular points which presumably help not only drug addicts but also patients with alcohol problems during their protracted withdrawal. Research showed that patients described amongst others feelings of wellbeing and relaxation, peacefulness and harmony, reductions of anxiety and drug consumption. However, the craving for drugs and alcohol remained and the effects were only temporary [43]. 

Further studies also showed a positive effect on combined therapies. For example, auricular acupuncture combined with carbamazepine treatment showed good results for alcohol withdrawals and combined with psychotherapy it was effective for drug patients [81, 82]. 

Here, too, more randomized controlled studies with higher number of patients need to be performed in order to receive more scientifically significant evidence on the effect of auricular acupuncture. 

### 18.8. Epileptic Seizures

In the last 20 years epilepsy, and especially drug-resistant epilepsy, has been successfully treated with vagus nerve stimulation (VNS) [83]. 

He et al. [29] hypothesize that auricular acupuncture can also be a possible treatment for epileptic seizures just as the VNS therapy. Clinical trials showed that auricular electroacupuncture led to a decrease of frequency and severity of seizures. 

It is still unclear how the antiseizure effect works, but it is assumed that auricular acupuncture as well as VNS triggers more than one neuromodulatory mechanism which leads to an effective treatment [33].

He et al. [29] suggest two mechanisms for the success of auricular acupuncture. Firstly, the activation of the NTS leading to a suppression of the affected parts in the brain and secondly on the assumption of Granata et al. [84] that epilepsy might be caused by immune-mediated processes, the activation of the cholinergic anti-inflammatory pathway [84].

Recent studies on trigeminal nerve stimulation (TNS) have also been successful in the treatment of epileptic seizures [85–87]. 

### 18.9. Smoking Cessation

In the last decades auricular as well as body acupuncture had been a very common method to help people to lead a life without tobacco. White et al. [88] published a large review of smoking cessation both in auricular as well as in body acupuncture. Although this study showed that the effects of acupuncture were not better than other methods of smoking cessation, the authors pointed out that there is evidence of the effect of acupuncture and further bias-free studies need to be conducted in order to prove these effects. A new method of auricular electro acupuncture, called “Smokex-Pro,” has been previously developed. A recently conducted study showed that after the treatment 47.9% of the patients stayed abstinent for at least 2 years [89]. 

### 18.10. Summary of Recent Studies

These aforementioned studies have shown that auricular acupuncture can be used in many fields of medicine and there is evidence of its effect. However, the lack of information, the small sample size, and the difference in study parameters give most of the studies a poor quality and make them difficult to compare. For more significant evidence, further investigations need to be conducted. 

## 19. Laser in Auricular Acupuncture 

### 19.1. Definition and History

The word “Laser” is an acronym for “Light Amplification by Stimulated Emission of Radiation.” The properties of laser such as its extreme monochromaticity, its polarisation, and its high coherence make the laser so unique and versatile [90]. 

### 19.2. History of the Laser

In 1917, Albert Einstein laid the foundation of the laser technology based on the principle of stimulated emission. Also based on this principle Charles H. Townes invented in 1954 the first MASER—short for microwave amplification by the stimulated emission of radiation—for which he received (with Alexander M. Prokhorov and Nikolai G. Basov) the Nobel Prize in 1964. In 1958, Townes and Arthur L. Schawlow published their study “Infrared and optical maser” which was used as a basis for the construction of the first laser in 1960 by the physicist Theodor Maiman. A development of various kinds of lasers ensued [91]. 

### 19.3. Laser in Medicine

In the early 1960s a Ruby laser was introduced into medicine for the photocoagulation of the retina. Due to severe side effects, the treatment was discontinued. In the late 1960s, an Argon laser was developed for the use of detached retina treatment. 

Today, laser therapy is used in many fields of medicine apart from ophthalmology also in dermatology, otolaryngology, dental medicine, general surgery, and vascular surgery. Depending on the aim of the treatment lasers can be of high intensity or low intensity. 

High-intensity (level) lasers have the ability to cut, destroy, or cauterize tissues due to their thermal effect whereas the effect of low-intensity (level) lasers (LLL), also called “soft laser” or “cold laser,” is believed to be caused by the interaction of electromagnetic radiation with the tissue. It has been shown to be effective in wound healing, musculoskeletal pain, and rheumatoid arthritis as well as in laser acupuncture [90, 91]. 

### 19.4. History of Laser Acupuncture

Between 1970 and 1972 reports on successful therapies of hypertension and asthma with laser body acupuncture in the USSR were published [92]. 

The Canadian Friedrich Plog suggested the use of laser in body acupuncture already in 1973. In 1979, the Chinese surgeon Zhou started using laser acupuncture instead of needle acupuncture and acupressure for anaesthesia successfully. First studies on laser auricular acupuncture have been reported in 1984 conducted by Seitz and Kleinkort [93]. Different laser instruments have been developed over the years. 

**Figure 13:**
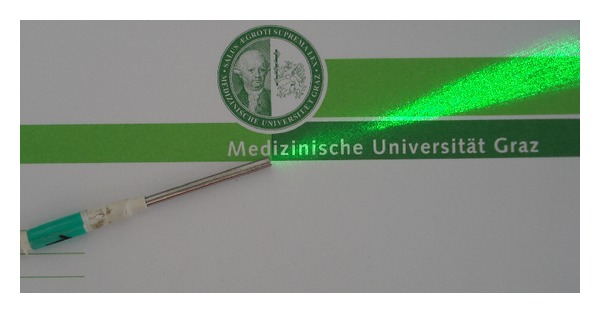
Laser needle for acupuncture.

In 2001, the first “laser needles” (Figure 13) were invented at the University of Paderborn (Germany). The first scientific investigations were performed at the Medical University of Graz. For the first time it was possible to stimulate different acupuncture points at the same time [94–97]. 

### 19.5. Types of Laser in Auricular Acupuncture

Low-level lasers (LLL) are the most commonly used lasers in body acupuncture as well as auricular acupuncture. They are defined by wavelengths between 300 nm and 10,600 nm, power densities between 10^−2^ W/cm^2^ and 10^0^ W/cm^2^, and energy densities between 10^−2^ J/cm^2^ and 10^2^ W/cm^2^. The wavelength of a laser is defined by its laser medium which can be gaseous, fluid, solid, or semiconducting [98]. 

In laser acupuncture the most frequently used lasers are gas lasers, mainly the Helium Neon (HeNe) lasers. In recent years, semiconductor lasers such as Aluminium Gallium Arsenide (AlGaAs) lasers or Gallium Arsenide (GaAs) lasers have become more popular in laser acupuncture. 

Most recently, ultralow level lasers (ULLL) have also been used in auricular acupuncture.

#### 19.5.1. Helium Neon Laser

The Helium Neon (HeNe) laser, a low-level laser, was one of the first gas lasers. The medium of the laser is a mixture of Helium and Neon. In laser acupuncture, HeNe lasers have a wavelength of 632.8 nm [99]. 

#### 19.5.2. Argon Laser

The Argon laser is also a low-level and gas laser. In laser acupuncture, the wavelength of an Argon laser is usually 514 nm [73]. 

#### 19.5.3. Aluminium Gallium Arsenide Laser

The Aluminium Gallium Arsenide (AlGaAs) laser is a semiconducting low-level laser. In laser acupuncture, the standardized wavelength is 780 nm [99]. 

#### 19.5.4. Gallium Arsenide Laser

The Gallium Arsenide (GaAr) laser is also one of the most commonly used semiconducting low-level lasers. Its standardized wavelength is 940 nm in laser acupuncture [99]. 

#### 19.5.5. Ultralow-Level Laser (ULLL)

Recently, there have been also studies of ultralow-level lasers (ULLL) in auricular as well as body acupuncture. The output power of an ULLL is significantly smaller than of an LLL; consequently, the energy density and the power density are also a lot smaller [65, 100, 101]. 

### 19.6. “Laser Needles” 

**Figure 14:**
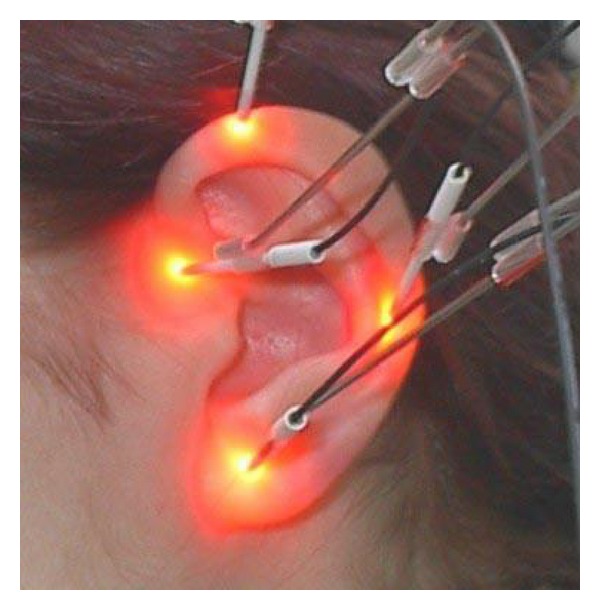
Laser needles. Modified from [97].

In 2001, the first “laser needles” were invented at the University of Paderborn in Germany, with the trade name Laserneedle (Figure 14). Since then, many new developments of these laser needles evolved. This semiconducting laser instrument is equipped with up to 10 laser needles and has the ability to work on different wavelengths, mainly red (685 nm), infrared (788 nm), and violet (405 nm) [95]. 

### 19.7. Classification and Safety of Low-Level Lasers

According to the European Norm (EN 60825-1), low-level lasers are classified as “3R” lasers equivalent to the old classification “3b,” meaning that radiation can be a risk of serious damage to the eye. To avoid this risk, the patient and acupuncturist should wear special glasses during the treatment. Laser as well as the treatment room must be marked specifically [10, 102].

## 20. Technical Parameters

### 20.1. Wavelength

The wavelength is one of the most important technical parameters in laser acupuncture. Its unit is expressed in nanometers (nm). A laser can have a wavelength spectrum between 240 nm and 3,000 nm. In laser acupuncture the lasers have wavelengths between 405 nm and 904 nm (Table 2). Lasers with wavelengths over 785 nm are infrared lasers in other words invisible light lasers. The wavelength is not only responsible for the coloring of the laser light but also how deep the laser light penetrates through the skin. For example, the red laser light with a wavelength of 685 nm has a penetration depth of about 4 cm [95, 103, 104].

### 20.2. Output Power

The output power is the power level of the laser light. Its unit is given in milliwatts (mW). In laser acupuncture, the applied dose depends on the output power of the laser. The higher the output power the higher the power density influences the depth of penetration. The designation of average output power is also very important especially when using a pulsed laser [95]. 

### 20.3. Power Density

The power density is expressed in terms of watt per cm^2^ (W/cm^2^) or milliwatt per cm^2^ (mW/cm^2^). It states the intensity of the laser beam and is in indirect proportion to the diameter of the laser beam [95]. 

Schikora [105] calculated that the power density had to be more than 1.3 W/cm^2^ for laser acupuncture to achieve the same effect as needle acupuncture. 

Calculation of the power density (mW/cm^2^) is as follows:

(1) $$\begin{array}{c} \text{Power}\;\text{Density}\;\left(\text{mW}/{\text{cm}}^{2}\right)=\frac{\text{Output}\;\text{Power}\;\left(\text{mW}\right)}{\text{Beam}\;\text{Area}\;\left({\text{cm}}^{2}\right)}. \end{array}$$

### 20.4. Energy Density

The unit of the energy density is watt-seconds per cm^2^ (Ws/cm^2^), which equals Joules per cm^2^ (J/cm^2^). The energy density is the treatment dose and states the amount of energy supplied per cm^2^ for the time of irradiation [95]. 

Calculation of the energy density (Ws/cm^2^)

(2) Energy  Density  (Ws/cm2)=Output  Power (mW)×Time (s)Beam  Area  (cm2).

### 20.5. Beam Diameter or Beam Area

Another important parameter is the beam area or the diameter of the beam (cm^2^). With one of these two parameters the energy density can be calculated if the output power and the time are stated. 

Calculation of the Beam area (cm^2^)

(3) $$\begin{array}{c} \text{Beam}\;\text{Area}\;\left({\text{cm}}^{2}\right)=\text{Diameter}\;\left(\text{cm}\right)\times 0.7854. \end{array}$$

### 20.6. Dose Range

According to the study of Litscher and Opitz [95], the dose range can be “from 0.001 J/cm^2^ (=Ws/cm^2^) to 10 J/cm^2^ (=Ws/cm^2^) and more.”

### 20.7. Continuous or Pulsed Laser

A laser can have pulsed or continuous waves. There is the assumption that pulsed laser light can “interfere with other pulsing biological phenomena” which would make a continuous waved laser a better option for laser acupuncture treatment. As mentioned previously, it is important to calculate the average output power when using pulsed lasers [95]. 

### 20.8. Time of Radiation

The time of radiation is also an important parameter in laser acupuncture. Most of the time it is in seconds (s). It is an important parameter for the calculation of energy density. The radiation time can last from 1 s to up to 100 s [37]. 

## 21. Important Study Parameters

According to Birch [106] studies on acupuncture respectively on auricular acupuncture should fulfil additional criteria to the already existing international standards of medical journals in order to achieve sufficient evidence. 

Most important parameters that should be considered in studies of auricular laser acupuncture are listed in the following. 

### 21.1. Research Question

The question of research needs to be clearly defined. On the basis of this research question, a proper study model shall be designed in order to achieve a meaningful result [106]. 

### 21.2. Randomized Controlled Studies

The use of randomized controlled studies has often been discussed in the literature. Still they are considered to be necessary for the efficiency of a study and stay as a gold standard [48, 106, 107]. 

### 21.3. Sample Size

The sample size in a study is also a very important parameter for the evidence of the effectiveness of auricular acupuncture. A smaller amount can be used in pilot studies however, in studies with more reference groups the amount of people has to be larger in order to give sufficient evidence. In most of the studies of auricular acupuncture, the number of treated patients is often too low [106]. 

### 21.4. Criteria

The inclusion and exclusion criteria need to be accurately defined and strictly executed [106]. 

### 21.5. Acupuncturist

Acupuncturists need to have very good qualifications which have to be stated in detail in the study. If there is more than one acupuncturist involved in the study then they should receive special training in accordance with the study. Particularly in auricular acupuncture with needles the points have to be needled correctly otherwise the results have a higher chance of being distorted. However, the risk of falsified results decreases by using a laser [106]. 

### 21.6. Nomenclature and Location

Due to the different nomenclatures in auricular acupuncture, the name and the exact location of the points need to be stated clearly in studies. 

### 21.7. Description of the Acupuncture Proceeding

The description of the proceeding has to include detailed information on the treated points, the system (e.g., Chinese or Western), the material used, the number and frequency of treatments, and the methods such as manual needling, electrical stimulation or laser stimulation. Studies on electroacupuncture and laser acupuncture need to provide proper information on technical parameters [106]. 

### 21.8. Period of Treatment

Another important parameter for the quality of a study is the time period of the treatment and the follow-up. Birch [106] suggested a time period of at least 3 months, preferably one year. The length of periods can vary depending on the pathological conditions; longer durations are also suggested for studies on drugs and alcohol abuse [108]. 

### 21.9. Number of Treatments

The amount of treatments and the time between treatments are very important parameters for reproducible studies in laser auricular acupuncture [109]. 

### 21.10. Reference Groups

Birch [106] recommended that the acupuncture treatment should always be compared to 3 different groups. The acupuncture treatment should be compared to sham acupuncture group, a group receiving standard medical therapy as well as a group which does not receive any treatment. Most of the studies compare auricular acupuncture to sham treatment groups or standard medical treatment groups. 

### 21.11. Sham Acupuncture versus Placebo Acupuncture

Birch [106] believes in most cases sham acupuncture with needles is used under wrong assumptions. Sham treatment is often mistakenly equated to placebo treatment. The chosen sham acupuncture points are not necessarily used for treatment of the medical condition that is investigated; however, they can still have an impact on the body when they are needled. 

In the study of Irnich et al. [110], sham laser acupuncture was conducted by deactivating the laser irradiation but not the red light or acoustic sound. In this case, sham laser acupuncture cannot be counted as a placebo treatment. It can only be treated as a placebo treatment if the laser stays entirely switched off. This must be clearly stated in the studies. 

### 21.12. Additional Medication

Studies where auricular acupuncture is used as an adjunctive treatment and studies of certain pathological conditions necessitate additional use of medical treatment, for example, withdrawal symptoms, anxiety, pain, anaesthesia, or hypertension. Here, the medication needs to be documented accurately [106]. 

## 22. Human Skin

### 22.1. Optical Properties of the Skin

The optical properties of the human skin play an important role in laser acupuncture. The multilayered, inhomogeneous, and anisotropic structure of the skin determines the absorption and scattering of the radiation. 

Melanin and haemoglobin are the main substances responsible for the absorption of the laser light. Melanin, responsible for the pigmentation of the skin, is mainly situated in the dermis and haemoglobin mainly in the epidermis. Further substances such as bilirubin, carotene, lipids, cell nuclei, and filamentous proteins may also contribute to the absorption of the laser light. 

The scattering of the radiation is mainly caused by the filamentous proteins Keratin, which is found in the epidermis and responsible for the thickness of the skin, and collagen in the dermis. Melanosomes in the epidermis, cell nuclei, cell walls, and other structures can also have an influence on the scattering of the radiation. 

Due to the dynamic of these substances their influence on the penetration of the laser light varies among human beings. Just to name a couple of examples, the thickness of the skin starts decreasing at the age of 45; the difference in the pigmentation of the Caucasian and the black population is caused by the different concentration of melanin [103, 111, 112]. 

### 22.2. Penetration of the Laser Light

The optical properties and the wavelength are the two main factors for the penetration of the laser light. It is believed that in body acupuncture the nociceptive structures lie 2-3 cm underneath the skin's surface. To achieve this depth, the wavelength must be between 605 nm and 850 nm which can be reached by red and infrared lasers [103, 105]. 

Recent studies with violet lasers have shown an effect on the blood flow velocity in the basilar artery by stimulating body acupuncture points. The penetration depth of the violet laser with a wavelength of 405 nm is about 1 mm [94]. 

Esnouf et al. [113] conducted a study evaluating the penetration depth of an Aluminium Gallium Arsenide laser with a wavelength of 850 nm. It showed that “most of the laser radiation was absorbed within the first 1 mm of the skin.” With this new finding it can be assumed that a violet laser can have the same effect as red or infrared lasers. Due to its low penetration depth the violet laser might even be the laser of first choice in auricular acupuncture since the penetration depth on the auricle does not necessarily require the same depth as in body laser acupuncture.

### 22.3. “Deqi” Sensation

In TCM the “Deqi” sensation is a signal to confirm the correct positioning of the needle in body acupuncture. “Deqi” can be seen as a benchmark of efficient acupuncture. It has been described as a feeling of numbness, prickling, flowing, or heaviness around the acupuncture point. Acupuncturists can make the “Deqi” sensation more perceptible by rotating or moving the needle back and forth during the therapy. In auricular acupuncture patients have described the “Deqi” feeling as a warm sense on the ear during treatment [45, 114]. 

Recent studies on body acupuncture suggest that “Deqi” sensation can also be achieved through laser acupuncture. In the studies the patients identified the “Deqi” sensation as a heavy sense or an electrical current or a feeling of an ant bite [94, 115]. 

## 23. Methods of Verification

In the last years, different noninvasive bioengineering methods have been used for verifying laser acupuncture. 

Methods for the measurement of the *peripheral effect* are

- Laser Doppler Flowmetry (LDF) and
- Laser Doppler Imaging (LDI).

The *cerebral effects* can be measured with

- multidirectional Transcranial Ultrasound Doppler Sonography (TCD),
- cerebral Near-Infrared Spectroscopy (NIRS), and
- functional Magnetic Resonance Imaging (fMRI).

### 23.1. Laser Doppler Flowmetry (LDF)

Laser Doppler flowmetry is a method to measure changes in the microcirculation. Flux is the main parameter and is defined as the product of average velocity and concentration of red blood cells in superficial vessels. This technique uses the Doppler effect—also called Doppler shift—which is based on the shifting of the light caused by moving blood cells [104, 116]. 

### 23.2. Laser Doppler Imaging (LDI)

This method is also based on the principle of the Doppler effect. Hereby, the reflected light acquires data that are transformed into a color-coded picture displaying the distribution of tissue perfusion [104]. 

### 23.3. Multidirectional Transcranial Ultrasound Doppler Sonography (TCD)

**Figure 15:**
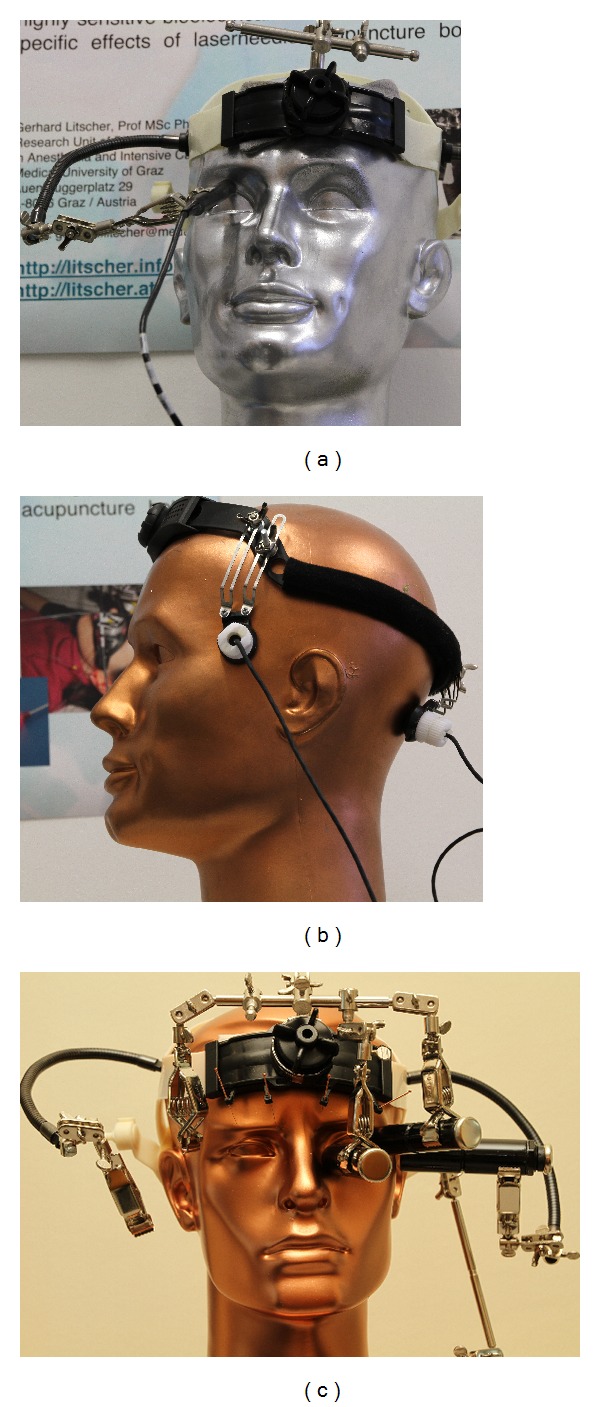
“Windows of the brain”: transorbital ((a), (c)), transnuchal ((b) right), transtemporal ((b) left, (c) right). Modified from [104].

The TCD is a relatively new recording technique in acupuncture which is able to measure the blood flow velocity in the middle cerebral artery, the anterior cerebral artery, the posterior cerebral artery, the supratrochlear artery, and the ophthalmic artery. The TCM Research Center in Graz developed a helmet for transcranial ultrasound investigations where the probe holder construction was situated at the “windows to the brain,” meaning transnuchal, transtemporal, and transorbital (Figure 15) [104]. 

### 23.4. Functional Magnetic Resonance Imaging (fMRI)

With this technique, it is possible to measure metabolic and circulatory changes of the brain. Hereby the fMRI uses the magnetic properties of oxygenated and deoxygenated haemoglobin to identify changes in their concentration in the blood [104]. 

### 23.5. Near Infrared Spectroscopy (NIRS)

NIRS is a noninvasive spectroscopic technique that measures the functional activities in the brain by using an optical window in the near-infrared light spectrum. The spectral range must be between 630 nm and 1300 nm, so the light can get through the cranium to investigate the metabolism in the cerebral cortex [104]. 

### 23.6. Bispectral Index (BIS) and Electroencephalogram (EEG)

The EEG is a method to measure electrical brain activities. In auricular acupuncture, EEG is mainly used to examine the activities of the cerebral cortex. 

The BIS is an important numerical descriptor of the EEG which is mainly used in anaesthesia [30, 104]. 

## 24. Advantages 

Taking into account that acupuncturists handle the laser equipment with care and responsibility, the advantages opposite the disadvantages in laser auricular acupuncture are outbalanced. 

### 24.1. General Advantages

Auricular acupuncture is often used as an adjunctive treatment, for example, during withdrawal therapies or for pain relief. It has shown that patients treated additionally with auricular acupuncture required less medication which further leads to a reduction of side effects and secondary diseases and consequently results in a decrease of costs [54–57, 72, 74]. 

Auricular acupuncture therapy does not require an inpatient treatment. Patients can be treated as outpatients which would further lead to a decrease of inpatients following a reduction of costs. 

### 24.2. Comparison to Auricular Acupuncture with Needles

The laser treatment being a noninvasive method is one of the biggest benefits. Patients with fear of needles as well as children of all ages can be treated. The low risks of local bleeding and infections such as perichondritis and chondritis disappear entirely [99]. 

In studies of auricular laser acupuncture a placebo treatment can be simulated easier than in studies of acupuncture with needles. 

Auricular acupuncture with laser has also the advantage that it takes less time than with needles [37]. 

### 24.3. Comparison to Body Acupuncture with Laser

Auricular acupuncture has two main advantages versus body acupuncture in needling as well as in laser treatment; on one side auricular acupuncture is easier to apply because the patients do not need to get undressed and on the other side the stimulation is not as sophisticated as in body acupuncture [59]. 

## 25. Disadvantages 

Risk of serious damage to the eye can be reduced drastically or even erased by cautious use of the laser instrument and also the wearing of safety glasses during the treatment.

## 26. Recent Studies and Their Quality

Not many studies have been published up to now. This is no surprise considering that auricular acupuncture has only found its way into medicine in the last 60 years and laser therapy only in the last 40 years. By using the keywords “auricular” and “acupuncture” and “laser” on PubMed as few as 28 publications were found. The first paper was published in the year 1982. Out of these 28 publications, 17 studies were published in English, 6 in Russian, 3 in Chinese, and 2 in Italian. 

Only 14 publications investigated specifically the effects of auricular acupuncture with laser. In the following 10 of these studies will be discussed in detail; the remaining 4 studies were either in Russian or Chinese and were not taken for further evaluation. 

With the exception of 2 studies, different subjects were investigated which made it difficult to compare the results of the publications with each other in order to find sufficient evidence of auricular acupuncture with laser.

### 26.1. Experimental Pain

King et al. [51] investigated on 80 healthy subjects whether laser auricular acupuncture can reduce pain or its threshold. Eighty healthy subjects with regard to the exclusion and inclusion criteria were chosen to take part. The subjects were randomly divided into 2 groups, an experimental group which received laser treatment on Shenmen, Lung point, Wrist point, and Dermis point and a control group which received sham acupuncture on the same points but without the laser being activated. Each point was stimulated for 30 s. For the stimulation of the acupuncture points, a continuous waved HeNe laser with a wavelength of 632.8 nm, an average power of 1 mW and a peak power of 1 mW was used. For the simulation of the pain an electric electrode was fixed on the left wrist of the subjects. There was no further information on the technical parameters such as power density, energy density, or the diameter of the laser beam. In 71% (29 subjects) of the experimental group there was an increase in the threshold of the pain whereas in the control group the increase was only 33% (13 subjects). 

The outcome shows evidence that laser auricular acupuncture can have an effect on pain. Further studies need to be conducted for more evidence; however, the missing technical parameters make a reproducibility of this study difficult. Studies on patients with real pain instead of experimental pain should also be investigated. 

### 26.2. Insomnia

In the Chinese study of Yao [117], 46 patients were treated for insomnia under the aspects of TCM. The treated auricular points were Shenmen, Endocrine point, Subcortex point, Brain point, Heart point, Kidney point, Spleen point, Stomach point, Liver point, and Kidney point. The laser instrument was a PU-1 semiconductor laser with a wavelength of 830 nm, an output power of 3 mW. The diameter of the laser beam was 2 mm. The irradiation time lasted for 1 minute. The treatment was once a day, 12 times with pauses of 5-to 7-day in-between therapy. The results showed that 69.5% of the patients (32 patients) were able to sleep for more than 7 hours, 28.3% of the patients (13 patients) were able to sleep between 5 and 6 hours. Auricular acupuncture had no effect on only one patient (2.2%). The total effectiveness was 97.8%. 

This outcome shows that auricular acupuncture can be effective in the treatment of insomnia. However, a description of the exact location of the points was missing and there was no information whether the patients received laser treatment on all of the mentioned points or only on certain points. The technical parameters did not include if it was a pulsed or continuous waved laser. No reference groups were selected. No inclusion or exclusion criteria were defined and the amount of people treated was too low to give sufficient evidence on this subject. 

Further studies need to be conducted for more evidence. Due to the lack of information, a reproducibility of this study is very difficult.

### 26.3. Acne Vulgaris

In a Chinese study of Sun [118], 68 patients were treated for acne vulgaris. These patients were randomly divided into a treatment group of 36 people who received laser auricular acupuncture and into a control group of 32 people who received body acupuncture with needles. For auricular acupuncture the points Lung, Spleen, Large Intestine, Sanjiao, Endocrine, Adrenal Gland, and Cheek were stimulated with an HeNe laser. Only 3 or 4 of these points were used for each treatment; however, there was no further information how often or in which combination these points were used and no description of the exact location of the auricular points. The HeNe laser had power density of 25 mW/cm^2^. The points were treated with this laser from a distance of 30–50 cm for 3–5 minutes per session. Altogether, the patients received 10 treatments. In the control group the patients were treated 10 times on different body acupuncture points with needles.

After the treatment 28 patients of the treatment group had no lesions left, in 6 cases 70% of the lesions were gone and only 2 patients had a cure rate of 30–70% of the lesions, whereas in the control group only 15 patients had a full cure of the lesions, in 14 cases 70% of the lesions were gone and only 30–70% of the lesions disappeared on 3 patients [118]. 

The results show evidence of the impact of auricular laser acupuncture. However, the exact location and combination of the auricular points, further technical parameters, for example, beam diameter or energy density, and a definition of exclusion and inclusion criteria were missing. Further studies with higher sample size and a third nontreatment reference group should be conducted for further proof of the effect of auricular laser acupuncture. The lack of information makes it again difficult to reproduce this study. 

### 26.4. Alcohol Addiction and Withdrawal

In recent years auricular acupuncture has been subject to many studies on alcohol addiction or alcohol withdrawals. So far two studies on different subjects in this field have been published on auricular acupuncture with laser. 

Zalewska-Kaszubska and Obzejta [73] investigated the possibility of auricular acupuncture as an adjunctive treatment of alcohol abuse and of its influence of the *β*-endorphin level in the plasma which is believed to have an influence on alcohol withdrawal. Fifty three patients were treated with a combination of auricular laser acupuncture and body laser acupuncture. The patients were chosen by exclusion and inclusion criteria. Out of these 53 patients only 15 patients finished the study. The patients were not divided into reference groups. 

The auricular points were stated in numbers 82, 83, 87, 51, and 55. According to Rubach [37], point number 82 is Point Zero, number 83 is the Anxiety point, 87 is the Stomach point, 51 is the Vegetative point I, and 55 is Shenmen. These points were stimulated with a semiconducting Argon laser with a wavelength of 540 nm, an output power of 100 mW, and a beam area of 0.05 cm^2^. The irradiation time lasted for 10 s per point. 

A spot on the neck vessel projection was stimulated by an HeNe laser with a wavelength of 632.8 nm, an output power of 25 mW, and a beam area of 0.2 cm^2^. The irradiation time was 5 min. There was no further information about the exact location of this spot. 

The whole treatment consisted of 4 sessions. Each session lasted 20 days. The auricle was stimulated every 2nd day and the neck vessel was continuously stimulated over a 20-day period. Inbetween the sessions there was a break of one week. For the valuation of the *β*-endorphin level, the patients gave blood before the first laser treatment and one day after each laser session. They also had to undertake the Beck Depression Inventory-Fast Screen (BDI-FS) after each session for evaluating their state of mind. 

The results showed an increase in the *β*-endorphin level and according to the BDI-FS a positive mood in the patients. The authors believe that an increase in the *β*-endorphin levels is linked to an increase in the mood and consequently a reduction of alcohol withdrawal [73]. 

This study gave sufficient information on the technical parameters of the lasers but insufficient information on the location of the stimulated auricular as well as body points. Although the results showed a positive outcome, the low number of patients which completed the study and the missing of reference groups call for further studies with better quality and information.

Trümpler et al. [119] investigated in their randomized controlled study the duration of alcohol withdrawal by comparing laser acupuncture with needle acupuncture and sham laser acupuncture on the auricle. Forty eight inpatients were chosen under certain criteria and were randomly divided into three reference groups. The points were chosen individually with the help of an electronic point finder. The most frequently stimulated points were—according to the nomenclature of Oleson—the Diaphragm point, the Cheerfulness point, the Insomnia point, the Sympathetic point, the Spleen point, the Laterality point, a Lung point, and Shenmen. A semiconducting laser with a wavelength of 830 nm (name: Modulus; product of schwa-medico, Inc., Ehringshausen, Germany) was used for the auricular laser acupuncture. No further technical parameters such as output power, diameter of the beam, or beam area were given. Each auricular point was stimulated for 60 s. In the laser sham acupuncture group the laser was inactivated. The needle acupuncture was performed with stainless steel needles to a depth of 1–3 mm for about 40 min. 

The treatment was applied daily until the end of withdrawal symptoms. Besides the acupuncture treatment, patients received Clomethiazole or in case of intoleration benzodiazepines on an individual basis. Other medications were kept unchanged for the time of the study. 

The outcome of this study did not show any difference of the withdrawal duration inbetween the reference groups. The needle acupuncture treatment seemed to have more efficiency than laser acupuncture treatment. However, there was no difference between sham and laser acupuncture. There was no information whether the sham acupuncture was applied with an entirely switched off laser or the laser irradiation was just deactivated. The authors see the cause for this insufficient result in the small number of patients, the different degrees of severity of diseases induced by long-term alcohol abuse, the different pharmacological management of withdrawal, and unequal provision of care [119]. 

For further improvement of this study's quality a non-treatment group should have been added as another reference group. Also, the combination of the individual auricular points should have been listed more precisely; maintaining to the standard use of the NADA might lead to a more meaningful result and better comparison for further studies. 

### 26.5. Chronic Allergic Dermatoses

Hou et al. [50] investigated the effects of auricular laser acupuncture on 35 patients suffering from eczemas, on 46 patients suffering from urticaria, on 41 suffering from fascial cosmetic dermatitis, and on 25 patients suffering from atopic dermatitis. The auricular acupuncture points Shenmen, Urticaria, and Subcortex were stimulated with a semiconducting AlGaAs laser each for 3 minutes. There was no further technical information of the laser. The whole treatment consisted of 2 sessions. Each session had a 5-minute treatment with intervals of 3-4 days inbetween. Medical treatment of antihistamine and corticosteroid was discontinued and a serum IgE level was determined before and after treatment. Only in the cases of eczema and urticarial, the IgE levels decreased after the treatment. The symptoms of all four dermatoses, however, improved significantly. 

Even though the results show evidence in the treatment of chronic dermatoses with auricular acupuncture, the deficiency of information, and the small amount of patients give this study a poor quality. 

For further evidence, a randomized controlled study with a larger number of patients, exact information of technical parameters, and definition of criteria as well as a longer period of treatment including a follow-up should be conducted. 

### 26.6. Laser Acupuncture for Adolescent Smokers

In this double-blind randomized study of Yiming et al. [62], auricular laser acupuncture was investigated for cessation of smoking on adolescent smokers. Three hundred thirty patients between the age of 12 and 18 years were divided into a laser acupuncture group and a sham acupuncture group. 128 adolescents of the laser acupuncture group completed the study and were treated on the auricular acupuncture points Ershénmén (probably Shenmen), Kó (probably Mouth), Fèi (probably Lung), and Wàibi. One hundred fourty adolescents of the sham acupuncture group completed the study and were treated on the same auricular points but without any radiation.

An HeNe laser with a wavelength of 632.8 nm, an output power of 2.5 to 3 mW, and a diameter of 1 mm was used for the laser stimulation. The auricular points were stimulated from a distance of about 1 mm for 60 s. The distance was chosen so that no pain or sensation could be felt. The treatment consisted of 12 sessions. Three sessions were given per week. 

The results showed no significant effect of auricular laser acupuncture compared to sham acupuncture. In both groups around 21% of the patients stopped smoking after the first treatment. A three-month follow-up showed a complete cessation of 25% of 101 patients in the laser acupuncture group and 26% of 107 patients in the sham acupuncture group [62]. 

The quality of this study was low. For a reproduction of this study, the nomenclature of the points and its localization should have been described according to the nomenclature of the WHO [14]. The details of the technical parameters were imprecise. It is also questionable whether with a distance of about 1 mm the laser acupuncture can have the same effect than by placing the laser needle directly on the skin. 

### 26.7. Animal Experiment: Microcirculation

Komori et al. [70] investigated in 40 rabbits the changes in microcirculation after auricular stimulation. The rabbits were randomly allocated into 4 reference groups with 10 rabbits each. The first group received needle acupuncture treatment, the second received near-infrared lamp irradiation, the third group received laser acupuncture stimulation, and the fourth group acted as control group and received no treatment. Stainless needles were used for the needle acupuncture treatment and a near-infrared lamp with a wavelength of 1540 nm and an energy density of 40 mW/mm^2^ were used for the near-infrared treatment. The irradiation time was 1 s. A semiconducting AlGaAs laser with a wavelength of 830 nm, an output power of 60 mW, an energy density of 39 mW/mm^2^, and a diameter of 1.4 mm were used for the laser acupuncture. The rabbit ear chamber method was used for the verification of the microcirculation. This method facilitates the observation of a single blood vessel via intravital microscopy as well as effects of different interventions on peripheral hemodynamics. 

The stimulated area on the rabbits' ears corresponds to the concha of the human auricle. The results showed a significant increase in the arteriolar diameter, the blood flow velocity and the blood flow rate after needle acupuncture, near-infrared lamp irradiation as well as laser acupuncture. Several studies have been conducted examining the relation of laser as well as needle acupuncture to the blood flow. Substance P—a neurotransmitter and calcitonin-related peptide—and Nitric oxide—responsible for vasodilatation—seemed to play an important role in this mechanism [70]. 

This study was of good quality and it proved that auricular acupuncture has an effect on the microcirculation. It can be considered to have an effect on diseases caused by poor peripheral blood flow. Further studies should be performed on this subject. 

### 26.8. Ultralow-Level Laser: Postural Instability

Bergamaschi et al. [100] investigated on 34 elderly patients the effects of auricular as well as body laser acupuncture with an ultralow-lever laser. The patients were divided into 3 reference groups. nine patients received auricular laser acupuncture, 9 patients received body laser acupuncture, and 16 patients received sham auricular acupuncture, hereby the laser stayed switched off. A semiconducting laser (product: BioliteLP020) with an output power of 0.03 mW was used. The irradiation was administered in 20 half-second flashes. The single point energy dose was about 0.3 mJ. The bioactive body acupuncture points were selected with a point finder. Instead of stimulating auricular acupuncture points auriculotherapy zones according to the Auriculoterapia Posturale secondo Scoppa method were stimulated. For the verification of the results, 4 balance tests were performed before treatment, right after treatment within 15 minutes of the first test, within 1 hour after the first test, and 3 days after the first test. The results showed an average improvement of 15% after the treatment and a long-term improvement of 5–10% 3 days after the treatment in body as well as auricular laser acupuncture. 

The results show slight evidence on the effects of body and auricular laser acupuncture. For further evidence, more studies on this subject need to be conducted with a higher amount of treated patients, a definition of exclusion and inclusion criteria plus the medication of the patients should be taken into account. 

### 26.9. Ultralow-Level Laser: Violet Laser Stimulation on Rats

Gao et al. [65] conducted a study testing the effects of a violet laser on heart rate, heart rate variability, and mean arterial blood pressure on anaesthetised rats by stimulating two body acupuncture points (Baihui (GV20) and Zusanli (ST36)) and the Heart point situated on the inferior concha of the auricle. The points were stimulated separately with a continuous waved laser (product: Conrad Electronic, SE) with a wavelength of 405 nm and an output power of 1 mW. The irradiation time lasted 2 minutes. The blood pressure was measured via a polyethylene catheter on the left common carotid artery connected to a blood pressure transducer and amplifier. The mean heart rate and the heart rate variability were monitored with an electrocardiogram (ECG) and measured before, during, and after the stimulation. 

The results showed changes in the heart rate on all acupuncture points especially after the stimulation of Baihui. The total heart rate variability changed insignificantly on all acupuncture points. The mean arterial blood pressure decreased slightly after stimulating the Baihui acupoint [65]. 

This study showed that a violet laser can have an impact on physiological neurovegetative parameters. However, there was no evidence that a violet laser had more effect on auricular acupuncture than on body acupuncture. Quite the contrary, the stimulation on Baihui showed more effect than on the Heart point. Further studies need to be carried out for further evidence.

## 27. Conclusion 

Recent studies have shown that auricular laser acupuncture can have an effect on the body. 

However, a comparison of the results is not given because of the small amount of studies, different subjects of research, and different study approaches. Studies in the past have often shown low quality due to insufficient and different levels of information. 

In order to achieve a higher grade of comparability the different technical and study parameters discussed previously have to be stated accurately. Furthermore, study designs of the same subject need to be reconciled with each other. To achieve meaningful and comparable results, further studies following these proposals need to be conducted. 

Recent conclusive studies of auricular acupressure, auricular electroacupuncture and auricular acupuncture, with needles should also be consulted for further studies on auricular laser acupuncture for example studies investigating the mechanism of auricular acupuncture. There is strong evidence that the influence on the autonomic nervous system through the stimulation of the ABVN can be one of its mechanisms [20, 26, 77]. 

By taking all these basic conditions into consideration, future studies on auricular laser acupuncture will be more significant and will show stronger evidence of its effect.

## Figures and Tables

**Figure 3:**
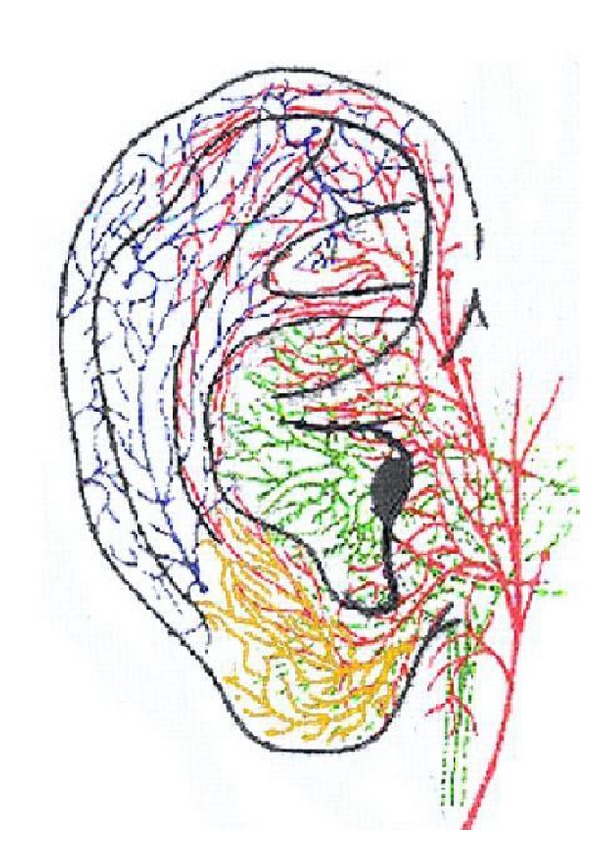
Green marks the branch of the vagus nerve, red marks the auriculotemporal nerve, blue marks the lesser occipital nerve, and yellow marks the greater auricular nerve. Modified from [20, 21].

**Table 1:** Innervation pattern (modified from [17]); auricle branch of the vagus nerve (ABVN), greater auricular nerve (GAN), auriculotemporal nerve (ATN).

	ABVN	GAN	ATN
Crus of helix	20%		80%
Spine of helix		9%	91%
Tail of helix		100%	
Scapha		100%	
Crura of antihelix	9%	91%	
Tragus	45%	46%	9%
Cymba conchae	100%		
Cavity conchae	45%	55%	
Lobule of auricle		100%	

**Table 2:** Wavelengths. Modified from [15].

Colour	Wavelength
Extreme violet	400 nm
Middle violet	420 nm
Blue-violet	440 nm
Middle blue (icy blue)	470 nm
Blue-green	500 nm
Green	530 nm
Green-yellow	560 nm
Sunlight	570 nm
Yellow	580 nm
Orange-yellow	590 nm
Orange	600 nm
Red-orange	610 nm
Light red	650 nm
Dark red	780 nm
